# Identification of Early Salt-Stress-Responsive Proteins in In Vitro *Prunus* Cultured Excised Roots

**DOI:** 10.3390/plants11162101

**Published:** 2022-08-12

**Authors:** Emma Sevilla, Pilar Andreu, María F. Fillat, M. Luisa Peleato, Juan A. Marín, Arancha Arbeloa

**Affiliations:** 1 Department of Biochemistry, Molecular Biology, Institute of Biocomputation, Physics of Complex Systems, Universidad de Zaragoza, 50009 Zaragoza, Spain; 2 Pomology Department, Estación Experimental de Aula Dei CSIC, Av. Montañana 1005, 50059 Zaragoza, Spain

**Keywords:** salinity, *Prunus cerasus*, in vitro root cultures, rootstocks, fructose 1,6-bisphosphate aldolase

## Abstract

Fruit-tree rootstock selection is a challenge under a scenario of growing environmental stresses in which the soil and climate are greatly affected. Salinization is an increasing global process that severely affects soil fertility. The selection of rootstocks with the ability to tolerate salt stress is essential. Excised root cultures may be an excellent experimental approach to study stress physiology and a predictive tool to assess possible tolerance. In this study, we show how protein changes in response to salt stress evaluated in excised root cultures of *Prunus cerasus* (moderate salt-sensitive cultivar) could be representative of these changes in the roots of whole plants. The 2D electrophoresis of root extracts and subsequent spot identification by MALDI-TOF/TOF-MS show 16 relevant proteins differentially expressed in roots as a response to 60 mM NaCl. Cytoplasmic isozyme fructose 1,6-bisphosphate aldolase shows relevant changes in its relative presence of isoforms as a response to saline stress, while the total level of enzymes remains similar. Ferredoxin-NADP^+^ reductase increases as a response to salinity, even though the measured activity is not significantly different. The observed changes are congruent with previous proteomic studies on the roots of whole plants that are involved in protection mechanisms against salt stress.

## 1. Introduction

Salinity is one of the main environmental factors limiting the productivity of agricultural crops because many cultivated plants cannot tolerate high concentrations of salt in soil. Unfortunately, the area of land across the world affected by salinity is rapidly increasing. Globally, 20% of cultivated land and 33% of irrigated land are affected and degraded by salt [1]. Plant roots are the primary site for sensing salinity and their plasticity is essential to achieve an adequate response in order to maintain functionality and absorb water and mineral nutrients despite the external water potential. Significant agronomical and social costs are associated with saline soils, and the goal of the studies on salt-responsive mechanisms is to either breed plants to resist salt tolerance or explore the biodiversity of the rich germplasms available in tolerant plants.

Under NaCl saline conditions, Na^+^ passively enters roots through non-selective cation channels [2]. Part of the Na^+^ is pumped out of the roots by the plasma membrane Na^+^/H^+^ antiporter pumps, with a considerable ATP cost. The remaining Na^+^ may be sequestered into vacuoles via tonoplast pumps or transported to the shoots through xylem [2]. Proteomic studies in several plant roots showed many changes involved in the homeostasis response to saline conditions, determining the phenotype of a plant tolerating salt stress [3,4]. Plant defense mechanisms include salt-stress perception and signal transduction, oxidative stress response, salt uptake/exclusion and compartmentalization, changes in gene expression and proteins, the cytoskeleton, cell wall dynamics, and carbohydrate and energy metabolism [3]. *Prunus* rootstocks show changes in growth, mineral content, and proline accumulation, in addition to genetic components in salt-stress-sensitive rootstocks compared to tolerant rootstocks [5].

As established in our previous studies, there are multiple preliminary responses of isolated root cultures to salt stress: (1) root length increment is inversely related to salt concentration; (2) starch deposition is correlated with salt concentration; and (3) proline exudation from the roots into the culture medium is also correlated with salt stress [6,7,8]. Moreover, the 60 mM NaCl concentration in culture media in vitro easily discriminates less tolerant species as *P. cerasus* or *P. dulcis* × *persica* from those that are more tolerant to salt stress, such as *P. insititia* or *P. cerasifera* × *munsoniana* [9]. Fruit trees are generally sensitive to salt stress, with differences depending on the species [10]. Interestingly, cherries, as species less tolerant to salt stress, have a similar response in aseptic root cultures in vitro compared to in the field [8,9,10].

Furthermore, 2-DE proteomic profiling has been used to study salt-stress responses in roots of different herbaceous plant species, revised by Zhao et al. [3]. However, there is limited information on the salinity stress responses of woody species, such as fruit trees and their rootstocks [5,11]. Current proteomic and transcriptomic studies have identified many proteins involved in salt tolerance, and this information is a valuable tool for studying adaptation to salt stress and the defining molecular markers of salt tolerance.

In this study, we show the suitability of in vitro studies of proteomic responses to salt stress in excised roots in the woody fruit tree species of *Prunus*.

In addition to studying the protein profile changes as a response to saline stress in *Prunus*, this study attempts to find a marker through in vitro root cultures due to their rapid response in comparison with field experiments. This could help to screen tolerance to salt in fruit rootstock breeding programs in order to adapt crops to the current climate change conditions.

## 2. Results

### 2.1. Growth in Presence of Different Salt Concentrations

Isolated root apexes (10 mm) of rootstock Masto de Montañana (*Prunus cerasus*) were cultured under aseptic conditions [6] for 15 days. Salt stress was applied by adding NaCl at 0 (control) or 60 mM to the culture medium.

**Figure 1:**
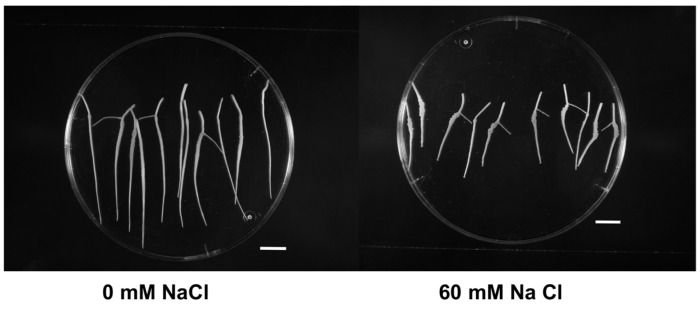
In vitro growth of *Prunus* excised roots of Masto de Montañana under different salt-stress conditions. The apexes in the figure correspond to day 15 (scale bar 10 mm).

Root fresh weight (FW), as a measure of root growth, decreased when salt stress was applied (Table 1, Figure 1). FW at 60 mM NaCl constituted only 46.4% of the control roots (0mM NaCl). Figure 1 shows the differences in the growth of excised roots of Masto de Montañana at day 15.

### 2.2. Proline Synthesis

Proline concentration in root tissues greatly increased at 60 mM of NaCl (391.0% compared to the proline concentration of the control roots, Table 1). These results indicate that root constitutive proline content significantly increased under salt stress, while root growth significantly decreased (Table 2). This indicates that Masto de Montañana, as in the field conditions, showed sensitivity to salt stress in isolated root cultures.

### 2.3. Identification of Differentially Expressed Proteins

**Figure 2:**
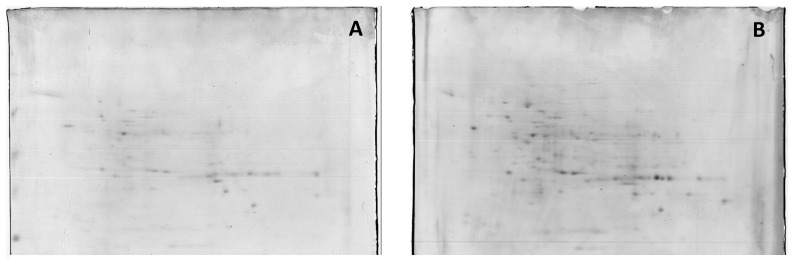
Representative 2-DE IEF-SDS PAGE protein profile maps of in vitro cultured whole-root extracts from Masto de Montañana. The roots were grown in 0 mM NaCl, (panel (**A**)) and supplemented with 60 mM NaCl (panel (**B**)). Figure 2 provides a general overview of the amount of proteins detected.

Proteins present in crude soluble extracts from the root cultures of Masto de Montañana were isolated by 2D electrophoresis, and changes in the 2-DE protein bands obtained from crude extracts at 60 mM NaCl versus 0 mM NaCl were studied. The total average numbers of detected spots (mean ± SD, n = 3) were 335 and 336 in 0 and 60 mM NaCl, respectively. Figure 2 shows 2-DE maps representative of the obtained replicas. Quantitative image analysis revealed the protein spots that changed their relative abundance and were differentially expressed. A 1.5-fold threshold value was selected in order to focus on protein identification in the most responsive proteins.

From 77 differentially expressed proteins (DEP) with a fold change of over 1.5, 35 were upregulated, while 42 were found downregulated. Six spots that significantly changed (*p* < 0.05) were successfully identified by MALDI-TOF, and ten relevant proteins with *p* > 0.05 were also successfully identified (Table 3).

**Figure 3:**
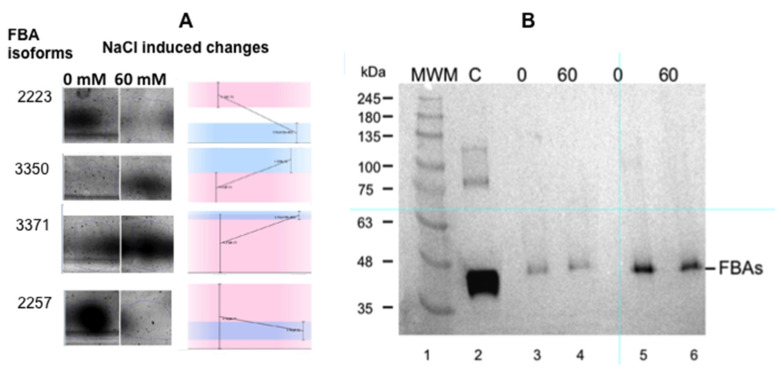
Panel (**A**): Changes in the expression of the different isoenzymes of fructose-1,6 bisphosphate aldolase (FBA) as a consequence of salt stress. (Masto de Montañana in vitro cultured excised roots). The small graphs indicate the changes detected by the gel reader. Panel (**B**): Western blot of the extracts used for proteomics studies (Anticytosolic fructose-1,6 bisphosphate aldolase (FBA8) from Agrisera^®^ (AS08294) were used at 1: 5000). Lane 1: molecular markers; lane 2: FAB control; lane 3: 0 mM NaCl cultured roots, 60 µg total protein extract; lane 4: 60 mM NaCl cultured roots, 60 µg total protein extract; lane 5: 0 mM NaCl cultured roots, 120 µg total protein extract; lane 6: 60 mM NaCl cultured roots, 120 µg total protein extract.

The most interesting change affected the relative amounts of different isoforms of the cytoplasmic isoenzyme of the fructose-bisphosphate aldolase (FBA): two isoforms increased, while two decreased (Figure 3A). Even though it is not possible to adjudicate the nature of the modification responsible for the different pI of the isoforms with this experimental approach, (redox modification or lysine methylation, for instance) [12,13], our results indicate that cytosolic isoform changes are involved in salt tolerance.

However, when the Western blots of the crude extracts of in vitro root growth in 0 and 60 mM salt were tested using FBA antibodies, the results show that there was no increase in total FBA in the salt-grown roots (Figure 3B). The antibody used was obtained against a cytosolic FBA, the FBA8 gene product. However, this antibody has a wide FBA crossreactivity due to the conserved sequences of FBAs. In our case, changes in the FBA isoforms associated with salt stress are remarkable, but is not possible to use the overall amount of FBA as marker (Figure 3). The expression of cytosolic AtFBA1, AtFBA2, AtFBA5, and AtFBA7 could not be detected in roots [14], so the FBA that was found presumably corresponds to FBA3, FBA4, FBA6, or FBA8 and their isoforms [14].

**Figure 4:**
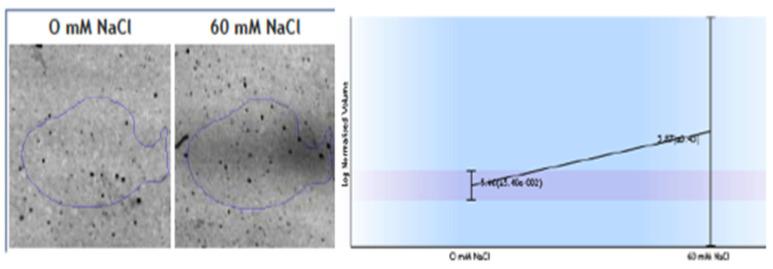
Changes observed in spots identified as root ferredoxin-NADP^+^ reductase (FNR) as a response to 60 mM NaCl. Diaphorase FNR enzymatic activity measured in crude extracts.

Other interesting protein changes as a response to salinity were observed; specifically, the upregulation of ferredoxin-NADP+ reductase (FNR) (Figure 4) and the downregulation of the V-type proton ATPase subunit B1. FNR diaphorase activity were tested in the crude extracts of the roots, and the samples obtained from roots grown in 60 mM NaCl exhibit only a slight increase (2.28 ± 0.03 IU in 0 NaCl and 2.5 + 0.1 IU in 60 mM Na Cl) in diaphorase activity (Figure 4).

Other differentially expressed proteins are involved in carbon and energy metabolism, such as the upregulation of glyceraldehyde-3-phosphate dehydrogenase and mitochondrial formate dehydrogenase. Enzymes related to amino acid metabolism, such as S-adenosylmethionine synthase 5 (cysteine and methionine metabolism), were enhanced, while there was a change in the isoforms or isozymes of D-3-phosphoglycerate dehydrogenase (involved in the plastidial phosphorylated pathway of serine biosynthesis) as a response to salt stress. (R)-mandelonitrile lyase 3, involved in *Prunus* cyanogenesis, which could play an additional role in nitrogen storage, was downregulated. Development and signalling proteins were also modified, especially the two proteins related to ubiquitin-mediated proteolysis: E3 ubiquitin-protein ligase UPL4 isoform X1 (downregulated) and heat shock cognate 70 kDa protein 2-like (upregulated). In the presence of 60 mM NaCl, the M20-dimer domain-containing protein involved in auxin metabolism decreased according to a reduced root growth in 60 mM NaCl. Finally, actin was upregulated, which was possibly caused by a potential ion compartmentalization in relation to salt-stress responses [15].

## 3. Discussion

Salt stress is a major environmental factor limiting plant growth and productivity. For plants challenged with salinity, roots represent the initial contact, and hence perception point; therefore, stress responses of the root system greatly influence the productivity of the whole plant [16]. For this reason, the in vitro screening of the responses of roots to salinity may be a fast and suitable experimental approach to select tolerant cultivars, which is especially useful in fruit trees.

Roots, as the first organs to detect changes in the soil water potential, have proven to be a good experimental model for detecting tolerance to abiotic stress such as salinity [17,18]. Our model based on excised roots culture [6,7,8,9] allowed for an early detection of salt-stress tolerance in the rootstocks of fruit trees through different characteristics, such as root growth, differences in the starch grains accumulated in root cells of the cortex, and the exudation of proline into the culture medium, as demonstrated in previous research [7,8,9]. In the present study, a decrease in fresh weight, increase in proline tissue concentration, and changes in proteins were observed in the roots of a species sensitive to saline stress—*Prunus cerasus*.

As expected, root fresh weight (FW), as a measure of growth (Table 1), indicated that the sensitive rootstock Masto de Montañana showed about half of the growth under salt stress as control roots (Table 1). This finding agrees with the relative increases in root length found in previous research [9], where Masto de Montañana showed a clear decrease in root length with 60 mM NaCl compared to other more tolerant species [9]. 

Tissue proline accumulation under salt stress has been correlated with salt tolerance [5,8]. In this study, a high increase in proline as a response to salt exposure occurred in the moderately sensitive cultivar, Masto de Montañana. This high increase may be due to the greater stress that 60 mM of NaCl produces in a sensitive species such as *P. cerasus* compared to more tolerant species such as *P. dulcis * × * persica* [5].

In this study, an in vitro approach was used to highlight whether cultured excised roots could detect similar responses in proteomics profiles compared to using roots from plants grown by traditional methods. Even though there are no proteomics studies on the responses of woody plants to salt stress, according to [19,20,21], woody plants have salt-tolerant mechanisms similar to those developed by non-woody plants. Several differential proteomics studies on the responses of herbaceous plant roots to salt stress have been published in recent decades [18,22,23,24,25]. The data are heterogeneous due to the different physiological fitness of the different species against saline stress as well as the different saline concentrations used or the experimental conditions, among other factors, such as the time of exposure. 

It is not the aim of this study to discuss the physiological mechanisms underlying salt tolerance or plant adaptation as there are several reviews that answer this question [19]. In vitro root physiology can be quite different from the physiology of roots in field conditions, but it is a valuable tool for screening early changes that may be indicative of salt tolerance [9]. The differentially expressed proteins detected in this study are proteins that were altered in roots of whole plants (Appendix A, Table A1), even though, and as indicated previously, there is a remarkable heterogeneity of data in the literature (due to the experimental conditions and the plant material itself), and it is very difficult to determine a common pattern of differentially expressed proteins that respond to salt stress.

The most striking results of our proteomics are the changes in isoforms of cytosolic fructose-bisphosphate aldolase (Table 3, Figure 3), which may indicate post-translational modifications in response to salt stress and the interconversion of isoforms to potentially adjust root physiology. The same diversity of isoforms turnover was previously described in aldolases of sugarcane [26], and this is found in other proteins in our study (Appendix A, Table A1). Two isoforms are downregulated, while other two isoforms are upregulated (Table 3). The nature of such modifications is difficult to identify, and several possibilities have been described, such as the modification of plant aldolase by redox modification of regulatory cysteines found by redox proteomics [12]. This mechanism was proposed as a potential mechanism to fine tune cellular energy metabolism in response to different levels of oxidative stress [27]. Van der Linde et al. [12] described the regulation of plant cytosolic aldolase functions by redox modifications, one of the forms showing the partial and reversible inactivation of enzyme activity.

There are many reports of increases in aldolase in response to certain stresses, and this has been widely identified in roots as derived from salt-stress responsive proteins [3,23]. Carbohydrate metabolism is essential for root function and stress tolerance; therefore, many studies associate changes in FBA with responses to salt stress [3,20,23,24,28,29,30,31,32]. The identification of the nature of each FBA isoform will provide a clue for understanding physiological adaptation to salinity.

Cytosolic fructose-bisphosphate aldolase not only plays a key role in glycolysis and gluconeogenesis, but also may play other important roles, such as modulating transcription factors imported to the nucleus [33]. Additionally, FBP aldolase can be involved in other essential responses such as ion vacuole compartmentalization. Barkla et al. [34] demonstrate that this enzyme can directly interact with and activate the V-ATPase subunit B, and aldolase was shown to stimulate V-ATPase activity in vitro by increasing its affinity for ATP, stimulating the ATPase present in tonoplast in ATP binding and hydrolysis activity, an important step for the transfer of salt into the vacuole, helping the plant cell eliminate the excess of ions Na^+^ and Cl^−^ of the cytoplasm. [25,35]. Moreover, it is interesting that, in our salt-treated samples, the V-type proton ATPase subunit B1 is downregulated, which was confirmed in other studies [35] (Appendix A, Table A1).

Glyceraldehyde-3-P-dehydrogenase was used as reference gene for salt-stress responses [36]. Two isoforms of plastidial D-3-phosphoglycerate dehydrogenase isozyme were identified, with interconversion as the response to salt stress. As in the case of aldolase, this may due to a thiol-based redox regulation that controls enzyme activity by switching the oxidation/reduction states of the Cys residues [37,38], and aldolase associates with an isoform of cytosolic glyceraldehyde-3-phosphate dehydrogenase, (upregulated in our proteomic analysis and in other references (Appendix A, Table A1). It appears to bind to the outer mitochondrial membrane, in a redox-dependent manner, leading to the binding and bundling of actin [38], also upregulated in our proteomics (Table 3).

In nonphotosynthetic cells such as roots, the FNR, sometimes called heterotrophic FNR, primarily functions in reverse to provide reduced ferredoxin for various metabolic pathways [39]. These pathways include nitrogen assimilation (nitrite reductase, glutamate synthase, sulphur assimilation, thioredoxin reductase signalling, oxidative stress response, and iron–sulphur protein biogenesis) [40]. Nevertheless, some studies suggested that FNR may play a crucial role in free radical balance in plants, being essential for development and accumulating as a response to stresses [41]. Our results indicate that FNR was upregulated under salt-stress conditions, with more proteins present (Table 3 and Figure 4), although we found almost no differences in the measurement of enzyme diaphorase activity.

## 4. Materials and Methods

### 4.1. Plant Materials, Culture Media and Treatments

The rootstock Masto de Montañana (*P. cerasus*) was micropropagated following previously described protocols [42]. Roots were aseptically cultivated under saline stress conditions as follows: Roots were washed in sterile distilled water and trimmed on a Petri dish placed over ruled paper to obtain 10 mm root tips. Eleven root tips per treatment (3 replicates) were cultured in 30 mL of liquid MS medium [43] with 3% sucrose and without growth regulators [9] and placed in the dark in an orbital shaker (90 rpm) in a culture room at 24 °C. Salt stress was applied by adding NaCl to the culture medium at 0 (control) or 60 mM, since in our previous study, using 0, 20, 60 and 180 mM NaCl, 60 mM NaCl, constituted a moderate stress with a clear response to salt without compromising the vitality of the tissue [9]. The total electric conductivity (E.C.) of the culture media at 25 °C was 6.0 and 12.5 dS·m^−1^, respectively. Root cultures were evaluated after 3 weeks, and root fresh weight was recorded using a precision balance. Roots were kept frozen until use.

### 4.2. Proline Determination

Proline colorimetric determination was followed according to the protocol of Bates et al. [44] and based on proline’s reaction with ninhydrin. The concentration of proline in root tissues was determined by triturating the frozen roots. Subsequently, 0.5 g of triturated roots was mixed with 2 mL of 3% sulphosalicylic acid. After centrifugation, the supernatant was mixed in a 1:1:1 ratio with ninhydrin acid and glacial acetic acid and incubated at 100 °C for 1 h. The reaction was stopped in an iced bath, and the chromophore was extracted with 4 mL toluene. The absorbance at 520 nm was determined in a BioMate spectrophotometer (Thermo Spectronic, Waltham, MA, USA). The method was calibrated for each determination with standard proline solutions (0–39 μg·mL^−1^). Statistically significant changes in FW and proline were determined using ANOVA.

### 4.3. Protein Extraction and Analytical Procedures

To analyse salt-response mechanisms at the protein level, root proteins were extracted from triplicated samples containing between 5 and 10 root tips (10 mm). Root tips were ground in a mortar and pestle into fine powders with 0.4 mg/g of PVPP (polyvinylpolypyrrolidone) for 8 min with the continuous presence of liquid nitrogen. The resulting powder was resuspended with 2 mL of precooled 90% acetone with 10% (*w*/*v*) TCA (trichloroacetic acid) and 0.1% of β-mercaptoethanol. The suspension was sonicated for 10 min at 4°C in a Branson^®^ Instrument at 45% of ultrasonic power, in ice bath and with special care to avoid heating. The resulting homogenate was incubated overnight at −20 °C before centrifugation (20 000× *g*, 15 min, 4 °C). The pellets were washed 3 times with precooled 90% acetone. Once the pellet was dried with N_2_ gas, it was stored at −20 °C for the different assays. 

### 4.4. Protein Quantification and Analytical Procedures

The protein content was quantified using the bicinchoninic acid method (BCA^TM^ Protein Assay Reagent Kit from Pierce). Then, 1D-Electrophoresis and immunoblotting of proteins were performed using SDS (sodium dodecyl sulfate)/12% polyacrylamide gels. For immunoblotting, proteins were electrophoretically transferred to PVDF (polyvinylidene difluoride membrane), with a 0.45 mm pore size in membranes according to Waters [45]. Anticytosolic fructose-1,6 bisphosphate aldolase (FBA8) from Agrisera^®^ (AS08294) was used at 1:5000. Blots were visualized and quantified as previously described [46].

FNR (ferredoxin-NADP+ reductase) activities of the crude extracts were spectrophotometrically measured using the dichlorophenolindophenol-dependent diaphorase assay, as described by Sancho et al. [47].

### 4.5. D-Electrophoresis, Gel Image and Statistical Analysis

Next, 2D electrophoresis was performed using the Proteomics Unit Service (CIBA, IACS, a ProteoRed ISCII member) and a modified protocol based on Krawitzky et al. [48].

The washed protein pellets were dissolved in 7 M urea, 2 M thiourea, 2% CHAPS (3-[(3-cholamidopropyl) dimethylammonio]-1-propanesulfonate) (*w*/*v*), 65 mM DTT (ditiotreitol) and 1% (*v*/*v* IPG buffer Sigma^®^, pH 3–10). The total protein concentration was determined with RCDC protein assay kit (Bio-Rad Life Science, Hercules, CA, USA). In this study, the crude extract isolated from the 0 and 60 mM NaCl-treated root tips was employed for 2-DE experiments and carried out using first-dimension isoelectric focusing (IEF) separation with a linear pH gradient 3–10. First-dimension separation was carried out in a Protean IEF (iso-electric focusing) cell (Bio-Rad Life Science, Hercules, CA, USA) following the manufacturer’s instructions. Then, 150 μg of protein per gels was loaded on 24 cm strips with an immobilized pH gradient (IPG) and a linear pH gradient 3–10 (GE Healthcare). The strips were developed at 90 µ/gel using the following settings: 10 min at 50 V, 1 h ramp up to 500 V, 1 h at 500 V, 2 h ramp up to 1000 V, 10 h ramp up to 10,000 V, and 2 h at 10,000 V. Prior to second-dimension SDS-PAGE, the strips were equilibrated for 15 min in DTT buffer (375 mM Tris–HCl pH 8.8, 6 M urea, 20% v/v glycerol, 2% *w*/*w* SDS and 130 mM DTT), followed by another 15 min incubation in iodoacetamide (375 mM Tris–HCl pH 8.8, 6 M urea, 20% *v*/*v* glycerol, 2% *w*/*w* SDS and 135 mM iodoacetamide). For the second dimension, strips were directly applied to 16% SDS-polyacrylamide gels, and 4 gels were simultaneously analysed on a Protean Plus Dodeca Cell (Bio-Rad Life Science, Hercules, CA, USA) at 1 W/gel until the bromophenol blue tracking front reached the end of the gel.

Immediately after 2D-DIGE, gels were individually scanned in a Typhoon Trio scanner (GE Healthcare, Chicago, IL, USA) and the Photo Multiplier Tube voltage was adjusted for maximum image quality with minimal signal saturation. Images were checked for saturation during the acquisition process using ImageQuantTM TL Software (GE Healthcare). Determination of protein spot intensity and correlation was performed using Progenesis SameS-pots v 4.0 software (Nonlinear Dynamics, U.K.). Statistically significant changes in protein spots were determined using ANOVA (*p* < 0.05; SameSpots Stats), and a cutoff of 1.5-fold change was established for further analyses. Gels were stained using a fresh solution of Colloidal Coomassie. In brief, gels were shaken for 1 h in an aqueous solution containing 16% ammonium sulphate, 3% (*v*/*v*) phosphoric acid, and 32% (*v*/*v*) methanol. Gels were transferred to a 6.6% Coomassie Blue G-250 methanolic solution for 3 h. De-staining was achieved by rinsing gels in water. For each treatment, gels were made from 3 independent root preparations, each from 6 different plants. The best gels for each treatment were selected for image analysis.

From all the data obtained in this study, at least three biological replicates were mixed for every sample treated. Then, statistical analyses, one-way ANOVA, and Duncan’s multiple range tests were performed with a 5% or 10% (as indicated) level of significance.

### 4.6. Protein Identification via MALDI TOF/TOF MS

The proteomics analysis was also performed in the Proteomics facility of Servicios Científico Técnicos CIBA (IACS-Universidad de Zaragoza). The Proteomics facility is a member of ProteoRed, PRB2-ISCIII.

In-gel digestion was performed as follows: Protein bands were excised from SDS-PAGE gels and washed with water, ammonium bicarbonate (25 mM NH_4_HCO_3_), and acetonitrile. Next, samples were reduced by incubation with dithiotreitol (10 mM) at 60 °C for 45 min and alkylated by incubation with iodoacetamide (50 mM) at room temperature for 30 min. Finally, proteins were digested using trypsin overnight at 37 °C with an enzyme:protein ratio of 1:10 (Trypsin Gold, Promega, Madison, WI, USA). Digestion was stopped by addition of 0.1% TFA (trifluoroacetic acid), and tryptic peptides were sequentially extracted with increasing concentrations of acetonitrile in water. Peptides were concentrated and desalted by passing them through ZipTip C18 columns (Millipore, Burlington, MA, USA) following the manufacturer’s instructions and eluting with 50%ACN (acetonitrile)/0.1%TFA.

Mass spectrometry analyses were performed as follows: The samples (0.5 L) and matrix (0.7 L saturated solution of alpha-Cyano-4-hydroxycinnamic acid (CHCA) in 50% ACN/0.1% TFA) were spotted onto an Opti-Tof 384-well insert plate (Applied Biosystems). MALDI-TOF MS was performed using a 4800plus MALDI-TOF/TOF (ABSciex) in the reflector mode with an accelerating voltage of 20 kV, mass range of 800 to 4000 Da, 1000 shots/spectrum and laser intensity of 2500. MSMS spectra were automatically determined on 20 of most intense precursors, with 1000 shots/spectrum and laser intensity of 3500. Spectra were externally calibrated using a standard protein mixture (4700 Calmix, Applied Biosystems, Waltham, MA, USA).

Protein identification was performed as follows: Proteins were identified with the search engine Mascot using the NCBI database against Viridiplantae (71310511 sequences). The search parameters used were: missed cleavage 1, fixed modifications carbamidomethyl (cysteines), and peptide and fragment mass tolerance, 0.2 Da and 0.3 Da, respectively. Individual ion scores > 49 were considered positive.

## 5. Conclusions

Changes in protein composition were observed in a *Prunus* rootstock grown under salt stress. An increase in proline, together with a decrease in root FW occurred with salt stress, and this is consistent with other studies [5,7,8,9,10]. In summary, proteomics studies were carried out on a rootstock that had undergone physiological and morphological changes associated with salt stress. The accumulation of proline as well as the lower FW of the roots were effects of salt stress. Proteomics studies show that the differentially expressed proteins were frequently described in the literature as responsive to salt stress. However, the amount of heterogeneous data shown by the literature on saline stress proteomics makes it difficult, both in this case and, generally, to compare the importance of each change. Another aspect to consider is the lack of research on the proteomics of fruit roots subjected to saline stress, and the current data for comparisons correspond to herbaceous plants. Aldolase isoenzymes interchange in response to salt stress and may play an important role in triggering responses. The most interesting physiological finding is the interconversion of isoforms of cytosolic fructose-bisphosphate aldolase detected in the salt-treated roots. The changes in the relative amount of the different isoforms of aldolase may be behind the physiological changes that occur in response to salt stress and may help to identify a molecular marker of tolerance to salinity in the future.

In vitro cultured excised roots are a promising tool to study stress responses in woody plants, which can contribute to a rapid and reliable screening tool for tolerant cultivars.

## Figures and Tables

**Table 1:** Fresh weight and proline concentration of roots (±s.e. of the mean of three replicates of 11 root apexes each) after 3 weeks of culture at 0 and 60 mM NaCl. (***) Indicates significant differences (*p <* 0.001).

Masto de Montañana	0 mM NaCl	60 mM NaCl	% of Control (0 mM)
FW (g)	0.6279 ± 0.0137	0.2911 ± 0.0127 (***)	46.36
Proline µmol/g	0.7068 ± 0.015	2.7634 ± 0.084 (***)	390.97

**Table 2:** One-way ANOVA of root FW and proline concentration after 3 weeks of culture at 0 and 60 mM NaCl. (***) Indicates significant differences (*p* < 0.001).

FW	Df	Sum Sq	Mean Sq	F value	Pr (>F)	
NaCl	1	0.17008	0.17008	325.2	5.56 × 10^−5^	***
Residuals	4	0.00209	0.00052			
**Proline**						
NaCl	1	6.344	6.344	580.9	1.76 × 10^−5^	***
Residuals	4	0.044	0–011			

**Table 3:** Differentially expressed proteins in in vitro cultured roots subject to salt stress. Fold change indicates the relative expression in 60 mM NaCl versus 0 mM NaCl in the culture media. Identification of the protein spots by MALDI-TOF/TOF with significant changes: *p* < 0.05 in the first six or *p* > 0.05 in the rest). The assigned protein is the best match found using Mascot. ↑ upregulated and ↓ downregulated proteins.

Spot No	Protein Identification	Fold Change	Functional Category and Biological Function	ANOVA (*p*)
3350	Fructose-bisphosphate aldolase cytoplasmic isozyme	1.8 ↑	Carbon metabolism; energy metabolism	0.013
2223	Fructose-bisphosphate aldolase cytoplasmic isozyme	2.3 ↓	Carbon metabolism; energy metabolism	0.002
3371	Fructose-bisphosphate aldolase cytoplasmic isozyme	1.7 ↑	Carbon metabolism; energy metabolism	0.045
1713	D-3-phosphoglycerate dehydrogenase isozyme plastidial 1	1.5 ↑	Amino acid metabolism	0.041
3366	Formate dehydrogenase (mitochondrial)	1.8 ↑	Carbon metabolism	0.041
1231	(R)-mandelonitrile lyase 3	2.3 ↓	Stress responses; signalling	0.050
3413	Ferredoxin-NADP^+^ reductase, root isozyme, plastidial	1.8 ↑	Energy metabolism; electron transport	0.2
2038	S-adenosylmethionine synthase 5	1.6 ↑	Metabolism; cysteine and methionine metabolism	0.1
2066	Actin	2.6 ↑	Cytoskelelum	0.4
2224	Glyceraldehyde-3-phosphate dehydrogenase	1.5 ↑	Carbon metabolism; energy metabolism	0.1
2257	Fructose-bisphosphate aldolase cytoplasmic isozyme	2.6 ↓	Carbon metabolism; energy metabolism	0.2
3358	Heat shock cognate 70 kDa protein 2-like	1.8 ↑	Stress responses; signalling; spliceosome	0.1
3407	V-type proton ATPase subunit B1	1.5 ↓	Energy metabolism; transport	0.1
2552	E3 ubiquitin-protein ligase UPL4 isoform X1	2.5 ↓	Gene processing; ubiquitin meditated proteolysis	0.2
968	M20-dimer domain-containing protein	2.4 ↓	Plant development; auxin metabolism	0.3
3414	D-3-phosphoglycerate dehydrogenase 1, plastidial	1.6 ↓	Amino acid metabolism	0.3

## Data Availability

Not applicable.

## Appendix A

**Table A1:** Some references in the literature concerning salt-responsive proteins in roots. Isoenzymes can exhibit different fold changes. ↑ upregulated and ↓ downregulated proteins.

Protein or Related Pathway	Fold Change in This study		Increased	Decreased
**Fructose-bisphosphate aldolase cytoplasmic isoforms**	1.8 ↑	1.7 ↑	[23,32,49]	[23,30]
	2.6 ↓	2.3 ↓		
Ferredoxin-NADP+ reductase, root isozyme, plastidial	1.8 ↑		[3,23]	
D-3-phosphoglycerate dehydrogenase plastidial isoforms 1	1.5 ↑	1.6 ↓	[50]	[50]
Formate dehydrogenase (mitochondrial)	1.8 ↑		[3]	
(R)-mandelonitrile lyase 3	2.3 ↓			[51]
S-adenosylmethionine synthase 5	1.6 ↑		[3,52]	[3]
Actin	2.6 ↑		[3,22]	[3]
Glyceraldehyde-3-phosphate dehydrogenase	1.5 ↑		[3,36,53,54]	[22]
Heat shock cognate 70 kDa protein 2-like	1.8 ↑		[55]	[54]
V-type proton ATPase subunit B1	1.5 ↓			[3,23,30]
E3 ubiquitin-protein ligase UPL4 isoform X1	2.5 ↓		[3,23]	
M20-dimer domain-containing protein →	2.4 ↓			[22]
